# The Influence of Mg Doping in α-Al_2_O_3_ Crystals Investigated with First-Principles Calculations and Experiment

**DOI:** 10.3390/ma18020407

**Published:** 2025-01-16

**Authors:** Yan Zeng, Haijun Fan, Haibo Guo, Kaiyong Tang, Zungang Wang, Siyuan Zhang, Mo Zhou, Li Fu, He Feng

**Affiliations:** 1 State Key Laboratory of NBC Protection for Civilian, Beijing 102205, China; yan-z@shu.edu.cn (Y.Z.); 13121370921@163.com (K.T.); zhigang7991@163.com (Z.W.); siyuanzhang68@163.com (S.Z.); weekend@mail.bnu.edu.cn (M.Z.); rainy5156@163.com (L.F.); 2 School of Material Science and Engineering, Shanghai University, Shanghai 200444, China; guohaibo@shu.edu.cn

**Keywords:** α-Al_2_O_3_:Mg, Al-Mg spinel, first-principles, doping mechanism, thermoluminescence

## Abstract

The influence of Mg doping in α-Al_2_O_3_ crystals is investigated in this article by first-principles calculations and formation energies, density of states, and computed absorption spectra. Three models related to Mg^2+^ substituting for Al^3+^ doping structures were constructed, as well as spinel structure models with varying aluminum-magnesium ratios. The formation energy calculations confirmed the rationality of the Mg_Al_V_O_ model, which means that Mg substitutional doping incorporating oxygen vacancies is most likely to form in crystals. The combined action of magnesium and oxygen vacancies introduced new defect energy levels in the bandgap. The calculated absorption spectra of the Mg_Al_V_O_ and Mg-rich spinel structures exhibited various color centers. The experimental absorption spectra and thermoluminescence characteristics of α-Al_2_O_3_:Mg and alumina-magnesium (Al-Mg) spinel crystal samples were tested. The thermoluminescence peak of the Al-Mg spinel was significantly stronger than that of the α-Al_2_O_3_:Mg crystal. The consistency between the model-calculated absorption spectra and the experimental results confirmed the theoretical predictions. Based on the experimental and computational results, the influence of Mg^2+^ substitutional doping in α-Al_2_O_3_ and the impact of the locally Mg-rich spinel on the optical and radiation performance of α-Al_2_O_3_:Mg crystals are elucidated.

## 1. Introduction

Alumina (Al_2_O_3_) is a well-known wide-bandgap insulator with a variety of point defects [1,2]. The performance of Al_2_O_3_ can be controlled by introducing kinds of color centers or defects. While some types of defects are inherent, some are produced by thermal reduction, irradiation with high-energy neutrons, electrons, or ions, and by adding impurities to the Al_2_O_3_ lattice [3,4]. For instance, α-Al_2_O_3_:C has been a mature radiation detection material. Due to the doping of carbon, the concentration of F and F^+^ centers in α-Al_2_O_3_ significantly increases, leading to a marked enhancement in its thermoluminescence (TL) and optically stimulated luminescence (OSL) properties [5,6]. Single crystal materials of α-Al_2_O_3_:C,Mg also exhibit excellent performance in radiation dose detection [7]. α-Al_2_O_3_:C,Mg crystals contain a large number of aggregated color centers, such as F^+^(Mg) centers. Under irradiation, this color center captures free electrons and transforms into the $F_{2}^{2+}$(2Mg) center, which can be used to store radiation information [8,9]. It allows for unlimited non-destructive repeated readings without the need for a chemical etching process, and the signal can be cleared for reuse, showing great potential and significant research value. However, the role of magnesium is not well understood, and its impact on the color centers in Al_2_O_3_ warrants further investigation.

Alpha-Alumina doped with magnesium (α-Al_2_O_3_:Mg) is a promising candidate for refractory materials [10], wear and corrosion-resistant coatings [11,12], ceramics [13], optical systems [14,15], and other applications across various fields. The nature of point defects within its crystal lattice significantly influences the material performance, making the study of color centers a crucial topic in these areas.

In α-Al_2_O_3_:Mg crystals, magnesium primarily exists in two forms: as Mg^2+^ ions substituting for Al^3+^ ions to form α-Al_2_O_3_:Mg and as spinel precipitates rich in magnesium. When these crystals are heat-treated in an oxidizing atmosphere, Mg^2+^ ions from the precipitates disperse into the α-Al_2_O_3_, creating a local environment rich in Mg^2+^ ions around the precipitates [14,16].

In the research on Mg substitution doping in Al_2_O_3_ crystal, it has been shown that the Mg particles replace Al sites in the alumina crystal lattice and form an oxygen vacancy around it, creating an [Mg]^0^ center with a corresponding center energy of 2.56 eV and an optical absorption band at 484 nm [17]. Additionally, Mg doping promotes the creation of more defect types in the crystal. For a single point defect, the concentration of F and F^+^ centers in the doped crystal increases significantly compared to undoped α-Al_2_O_3_ crystals. Mg ion doping also facilitates the formation of more stable F and F^+^ centers than in undoped crystals [15]. The absorption bands related to F and F^+^ centers appear at approximately 6.0 eV (~206 nm) and 4.8 eV (~258 nm), respectively. The observed energies of the bands in question align with those found in undoped crystals following irradiation, suggesting that Mg^2+^ ions in α-Al_2_O_3_:Mg crystals do not exert a substantial influence on these centers. Studies on the properties of color centers induced by thermochemical reduction in α-Al_2_O_3_:Mg crystals at high temperatures have shown that isolated oxygen vacancies and higher-order defects are more likely to form in α-Al_2_O_3_:Mg than in pure α-Al_2_O_3_ crystals. At 773 K, prolonged heat treatment can easily form higher-order aggregates such as $F_{2}^{2+}$ and $F_{2}^{+}$, with strong absorption bands at 2.87 eV (432 nm) and 3.69 eV (336 nm), which centers were perturbed by Mg. The experiment also observed photoconversion of $F_{2}^{2+}$ and $F_{2}^{+}$ centers. In crystals with high concentrations of $F_{2}^{2+}$ centers, electrons excited by 5.0 eV light are captured by $F_{2}^{2+}$ and $F_{2}^{+}$ centers, leading to the transformation of $F_{2}^{2+}$ centers into $F_{2}^{+}$ and, to a lesser extent, into F_2_ centers [14].

Meanwhile, localized Mg^2+^ aggregation may lead to the formation of alumina-magnesium (Al-Mg) spinel structures in the grown α-Al_2_O_3_:Mg crystals [14,16]. Al^3+^ on the tetrahedral site, due to its extra positive charge, can act as an electron trap, while Mg^2+^ on the octahedral site, due to the lack of a positive charge, can act as a hole trap [18,19,20]. Thus, it fulfills the conditions for the formation of multiple defect structures. The RL spectrum shows that MgAl_2_O_4_ has a broad band centered at 400 nm and a narrow band at 515 nm with a peak at 687 nm in the 230–750 nm spectral range, where the broad emissions around 710–750 nm and at about 650 nm are related to color centers associated with Mg vacancies. The emission band observed at 461 nm under excitation at 234 nm is attributed to the F center because the excitation band corresponds to the absorption band of the F center. However, based on the similarity of the F to F^+^ conversion process in Al_2_O_3_ [21], the excitation band at 261 nm is also attributed to the F^+^ center.

From the above literature, it can be seen that the role of Mg^2+^ ions in α-Al_2_O_3_ crystals and the formation of color centers are closely related to complex defect states in the crystal. To investigate the influence of Mg on α-Al_2_O_3_, we constructed three models for Mg^2+^ substituting for Al^3+^ doping structures and spinel structure models with varying Al to Mg ratios. These models were used to explore the significant role of Mg doping by analyzing the density of states (DOS) and calculating the absorption spectra combined with first-principles calculations. The absorption spectra and TL properties of α-Al_2_O_3_:Mg crystal samples prepared by the optical floating zone method and the Mg-Al spinel crystals were tested. The experimental results validated the rationality of the model construction. Based on the experimental and computational results, we elucidated the role of Mg^2+^ substitutional doping in α-Al_2_O_3_ and the influence of the Mg-Al spinel with Mg-enriched condition on the optical and radiative properties of α-Al_2_O_3_:Mg crystals. This study provides a reference for modulating defects or color centers in α-Al_2_O_3_ through Mg doping and optimizing its dosimetric performance.

## 2. Theoretical Method

DFT calculations were performed using the Vienna ab initio simulation package (VASP) [22]. The Perdew–Burke–Ernzerhof (PBE) functional within the generalized gradient approximation (GGA) [23] and hybrid exchange-correlation functional (HSE06) [24,25] were used to process the exchange-correlation, while the projector augmented-wave pseudopotential (PAW) [26] was applied with a kinetic energy cut-off of 520 eV, which converged well with the total energy of the system. Brillouin zone integration was sampled using a Γ-centered 6 × 6 × 6 Monkhorst–Pack K-point. All atomic positions were fully relaxed until the energy and force reached tolerances of 1 × 10^−5^ eV and 0.01 eV/Å, respectively. The dispersion-corrected DFT-D method was employed to consider the long-range interactions [27]. All graphs of the crystal structures were generated using the graphics software Visualization for Electronic and STructural Analysis (VESTA ver.3.5.8) [28].

**Figure 1:**
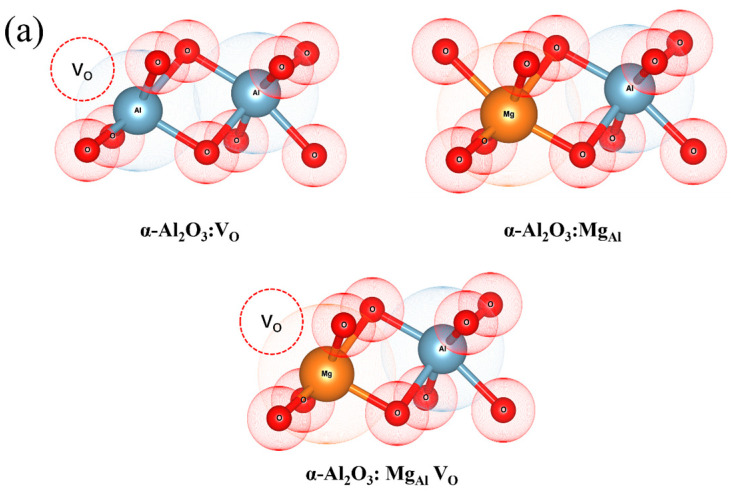

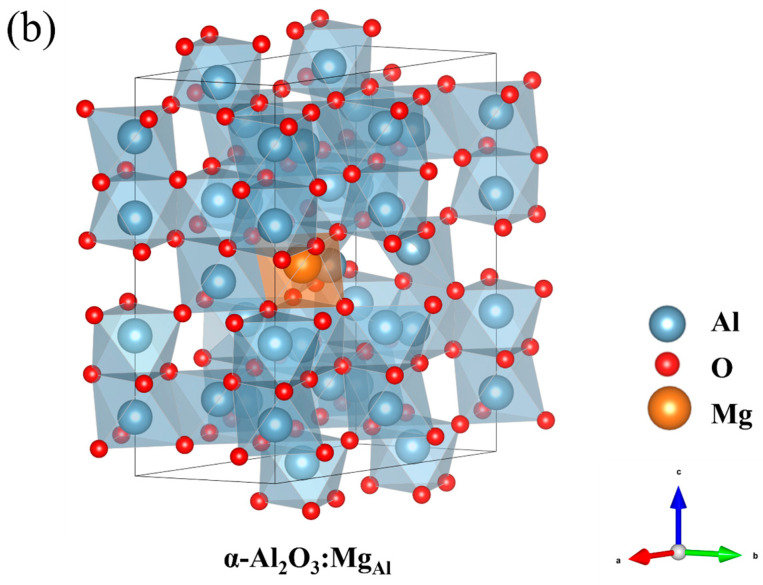
(**a**) The schematic diagrams of the doping positions of the V_O_, Mg_Al_, and Mg_Al_V_O_ structures; (**b**) schematic representation of α-Al_2_O_3_:Mg.

All models built in this study are based on the α-Al_2_O_3_ structure, which has a triclinic structure with the crystal symmetry of R-3c (No.167). The coordination numbers of the Al and O atoms in the crystal lattice are 6 and 4, respectively. The interatomic configurations depicted in Figure 1a encompass Mg substituting for Al sites (Mg_Al_), isolated oxygen vacancies (V_O_), and complex defects featuring both Mg substitutional doping and oxygen vacancies (Mg_Al_V_O_). In the composite defect models, vacancies are positioned at the nearest neighbor sites to the Mg doping locations. The supercell incorporated Mg in α-Al_2_O_3_, consisting of 60 atoms (Figure 1b). The other relevant properties for all structures were calculated after completion of the structural optimization.

**Figure 2:**
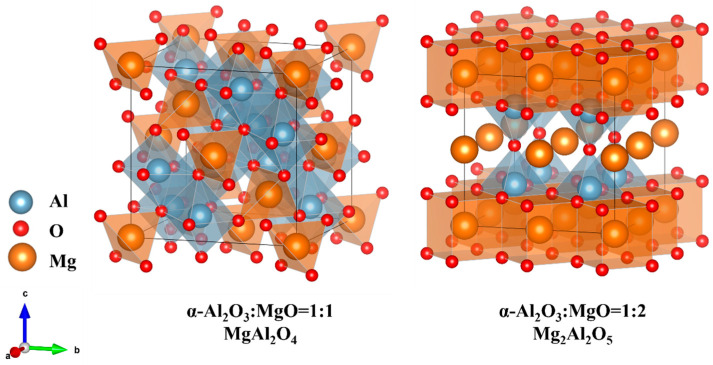
Schematic representation of the structure of MgAl_2_O_4_ with a 1:1 ratio of α-Al_2_O_3_ to MgO, and the structure of Mg_2_Al_2_O_5_ with a 1:2 ratio of α-Al_2_O_3_ to MgO.

Aluminum-magnesium (Al-Mg) spinel structures could be formed with 1:1 (MgAl_2_O_4_) or 1:2 (Mg_2_Al_2_O_5_) ratios of α-Al_2_O_3_ and MgO during the α-Al_2_O_3_:Mg growth process. MgAl_2_O_4_ is spinel-structured and crystallizes in the cubic Fd̅3m space group. Mg_2_Al_2_O_5_ crystallizes in the tetragonal P4/mmm space group. For this reason, we constructed the structure of the Al-Mg spinel with two common ratios, as shown in Figure 2. The valence electron states of the elements involved in the calculations are 3s^2^3p^1^ for Al, 2s^2^2p^4^ for O, and 2p^6^3s^2^ for the Mg atoms.

The defect formation energy $E_{form}$ depends on the Fermi energy level and chemical potential of the substance associated with the defect, and can be expressed as follows in Equation (1):

(1) $$E_{form}=E_{tot}\left(defect\right)-E_{tot}\left(perfect\right)+\underset{i}{\sum }n_{i}\left(E_{i}+\mu_{i}\right)\;$$

where $E_{tot}\left(defect\right)$ and $E_{tot}\left(perfect\right)$ are the total energies of Mg doping α-Al_2_O_3_ and perfect α-Al_2_O_3_ supercells, respectively. $E_{i}$ and $\mu_{i}$ correspond to the energies and chemical potentials of the individual atoms of the corresponding elements, ad $n_{i}\;$ is the number of atoms removed from or added to the supercell.

The chemical potentials of Al, O and Mg in α-Al_2_O_3_ are restricted by the following conditions:

(2) $$2\mu_{Al}+3\mu_{O}=E\left(Al_{2}O_{3}\right)$$

(3) $$\mu_{Mg}\leq E\left(Mg\right)$$

(4) $$\mu_{O}\leq \frac{1}{2}E\left(O_{2}\right)$$

(5) $$\mu_{Al}\leq E\left(Al\right)$$

(6) $$\mu_{O}+\mu_{Mg}\leq E\left(MgO\right)$$

where $E\left(Al_{2}O_{3}\right)$ is the total energy of the bulk α-Al_2_O_3_. Considering that the growth environment of the crystals is an air atmosphere, we only consider the defect formation energy under O-rich conditions in our calculations.

The optical properties are calculated based on the combined DFT with many-body perturbation theory in the G_0_W_0_ approximation [29,30]. The electron hole interaction is taken into account by solving the Bethe –Salpeter equation (BSE) [22,23,31]. The absorption spectra of the materials are calculated based on the real part *ε*_1_ (*ω*) and imaginary part *ε*_2_ (*ω*) of the dielectric function by the following Equation (7):

(7) $$\alpha \left(\omega \right)=\frac{\sqrt{2}\omega }{c}{\left(\sqrt{\varepsilon_{1}^{2}+\varepsilon_{2}^{2}}-\varepsilon_{1}\right)}^{\frac{1}{2}}$$

where *α*(*ω*) is the absorption coefficient of Al_2_O_3_:Mg, and c is the speed of light. Both the dielectric function and the calculation procedure are done with VASPKIT (ver.1.4.0) [29].

## 3. Experimental Method

α-Al_2_O_3_:Mg crystals were synthesized using the optical floating zone method under an air atmosphere. α-Al_2_O_3_:Mg crystals with Mg doping concentrations of 300 ppm were prepared. High-purity raw materials (99.999% pure MgO and Al_2_O_3_). The materials were sintered at 1500 °C for 8 h in air to produce polycrystalline rods. The crystal growth was performed using a floating zone method in a vertical double-ellipsoid mirror furnace, powered by a 6500 W Xe arc lamp (SciDre HKZ, Dresden, Germany). Single crystals, approximately 5 mm in diameter and 1 mm in thickness, were cut and polished on both sides for the subsequent measurements.

**Figure 3:**
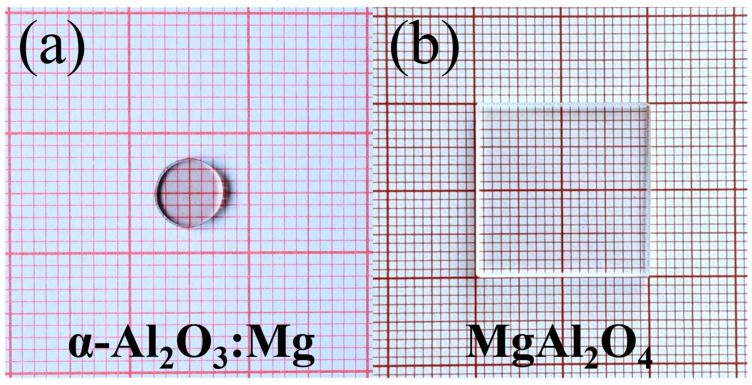
(**a**) Ф5×1 mm α-Al_2_O_3_:Mg single crystal wafer polished on both sides. (**b**) Single crystals of MgAl_2_O_4_ were grown by the Cz method, cut to 20 × 20 × 1 mm, and polished on both sides.

The MgAl_2_O_4_ crystals were synthesized with powders of MgO (99.999%) and Al_2_O_3_ (99.999%) as raw materials. The powders were combined and blended in a mixer for a period of 24 h to ensure thorough mixing of the ingredients. The resulting mixture was then packed into a mold and compacted. The pressed cylinders were transferred to a muffle furnace and sintered at 1100 °C for 12 h to form polycrystalline MgAl_2_O_4_. The growth of MgAl_2_O_4_ single crystals was carried out by the Czochralski (Cz) method in an air atmosphere. The sintered polycrystalline MgAl_2_O_4_ was put into an iridium crucible equipped with an intermediate frequency induction heating system Cz furnace, heated until the polycrystalline material was completely melted and held for a period. A series of subsequent crystal growth processes were conducted with the aid of an automatic control system, with a pulling rate of 2–4 mm·h^−1^ and a rotation rate of 5–10 r/min. Figure 3 displays photographs of the α-Al_2_O_3_:Mg and MgAl_2_O_4_ crystal samples. The grown crystals were cut to 20 × 20 × 1 mm and polished on both sides for the measurements.

XRD analysis was performed with an X’Pert3 Powder(Almelo, Netherlands) diffractometer with CuKa radiation. Data were collected with steps of 0.013° (2*θ*). Diffraction patterns of alpha-phase alumina were compared with reference to the JCPDS database. The absorption spectra were recorded at room temperature using a UV/visible/NIR spectrophotometer (HITACHI UH4510, Hitachi, Japan). TL measurements were conducted using the Risø TL/OSL DA-20 reader. The samples were irradiated at room temperature with a ^90^Sr/^90^Y beta source at a nominal dose rate of 0.05 Gy/s. The luminescence was detected by an EMI 9107QB PMT with a 7.5 mm Hoya U-340 filter. It was carried out at heating rates of 5 K/s.

## 4. Results and Discussion

### 4.1. Calculation Section

Building on predecessors’ research, the doping sites of Mg elements have been identified. When Mg is doped into the Al_2_O_3_ matrix, it primarily forms point defects by Mg^2+^ replacing Al^3+^ sites. Consequently, the model construction has focused on analyzing the effects of Mg substitutional doping, without elaborating on other sites. We further constructed complex defect structures related to Mg substitutional doping and analyzed them. Based on the role of oxygen vacancies in the formation of color centers, we established Mg_Al_V_O_ structures.

The formation energies of the aforementioned structures in their charge-balanced states are shown in Table 1. Only defect formation energies are discussed here, so the formation energy of the Al-Mg spinel is not mentioned. The formation energy of Mg substitutional doping is 3.368 eV. By introducing an oxygen vacancy in this structure, the formation energy becomes 2.189 eV, which is lower than the energy required for the formation of a single Mg substitutional structure. The formation energy for directly creating an oxygen vacancy in α-Al_2_O_3_ single crystals (α-Al_2_O_3_:V_O_) is 1.952 eV. This suggests that Mg substitutional doping structures incorporating oxygen vacancies are more likely to form in crystals.

Formation energy calculations verify the plausibility of the presence of an Mg_Al_V_O_ structure in α-Al_2_O_3_:Mg crystals. Because the proposed Mg_Al_V_O_ structure has not been previously documented, a rigorous examination of its rationality has been undertaken. This investigation entailed a thorough evaluation of the structural stability of Mg_Al_V_O_, as shown in Table 2. The parameters enumerated in Table 2 are pivotal in evaluating the structural stability. The outcomes of Young’s modulus (E) and shear modulus (G) demonstrate that the Mg_Al_V_O_ structure exhibits remarkable resistance to elastic deformation and shear deformation. Furthermore, the bulk modulus (B) and Vickers hardness indicate that the structure demonstrates enhanced resistance to volumetric change and plastic deformation. These calculations suggest that the Mg_Al_V_O_ structure possesses excellent structural stability.

**Figure 4:**
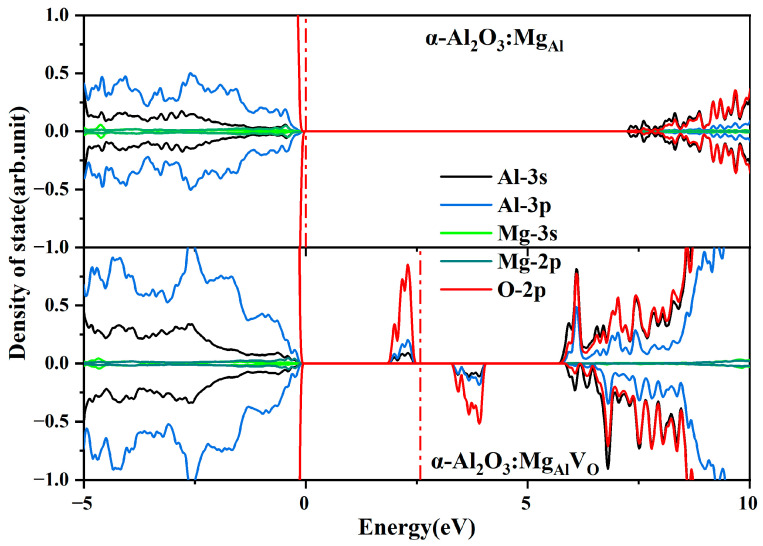
Atomic projected DOS of neutral Mg_Al_ and Mg_Al_V_O_. Zero energy is aligned to the top of the VBM. The red dotted line is the Fermi energy level.

And, we analyze it in comparison with the DOS of the single Mg-doped model structure on the HSE06 algorithm. Figure 4 presents the partial density of state (PDOS) distributions of Mg_Al_ and Mg_Al_V_O_. Both the valence band (VBM) and conduction band (CBM) of these point defects and complex defects are mainly generated by the electron orbitals of O-2p, Al-3p, and Al-3s. The bandgap of the Mg_Al_ structure is 7.18 eV, and for the Mg_Al_V_O_ structure, it is 7.34 eV, which is an increase of 0.16 eV relative to the Mg_Al_ structure. No gap states were observed in the Mg_Al_ structure, which deviated from our initial expectations. However, in the bandgap of the Mg_Al_V_O_ structure, one occupied energy level and one unoccupied energy level appear, both mainly contributed by the O-2p orbitals. Electron transitions from the occupied states to the empty states in the spin-down states can occur. It is important to note that the contribution of Mg to the orbitals is minimal in both single doping and complex defects. Electrons can transition from the occupied state to the spin-down unoccupied state. Thus, these gap states can act as electron traps, capturing electrons. The trap depth of the Mg_Al_V_O_ structure, calculated relative to the CBM, is 1.46 eV. The analysis suggests that Mg does not directly participate in the formation of trap energy levels. We hypothesize that the main role of Mg is to form defects by compensating the charge of the oxygen vacancies.

**Figure 5:**
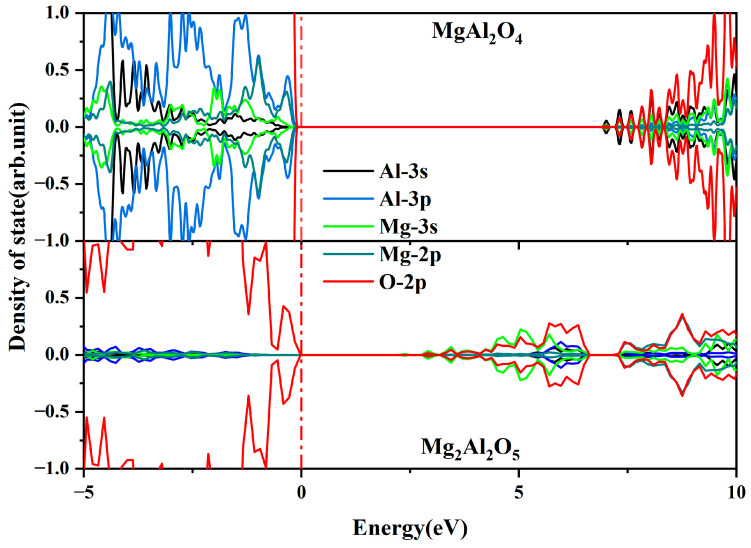
Atomic PDOS of aluminum-magnesium spinel structures with 1:1 or 1:2 ratios of α-Al_2_O_3_ and MgO. Zero energy is aligned to the top of the VBM. The red dotted line is the Fermi energy level.

By employing the same computational methodology, the PDOS for the Al-Mg spinel structures with 1:1 and 1:2 ratios of α-Al_2_O_3_ to MgO is illustrated in Figure 5. The bandgaps of these structures are 6.87 eV and 7.16 eV, respectively, which are close to the bandgap of Mg-doped α-Al_2_O_3_. This satisfies the prerequisite for the local Al-Mg spinel structure to potentially influence the color center performance of α-Al_2_O_3_:Mg crystals. Two spin-opposite occupied gap states are observed in the DOS of the Mg_2_Al_2_O_5_ structure. A significant contribution from the hybridization of Mg-3s and O-2p orbitals to the generation of gap states is noticeable. This phenomenon differs from several previously mentioned structures, and only the structure under Mg-enriched conditions exhibits a Mg contribution to the gap state. This indicates that when Mg locally aggregates to form an Al-Mg spinel structure, the resulting local Mg enrichment is conducive to creating defects within the crystal, promoting the formation of color centers.

**Figure 6:**
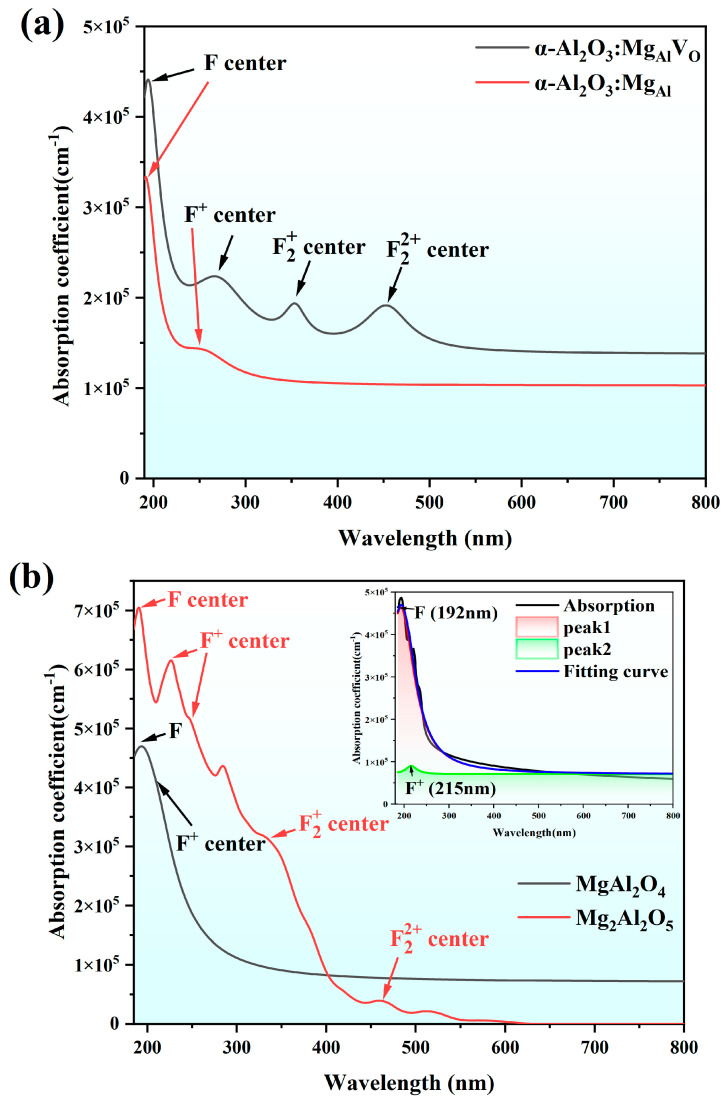
(**a**) Fitting of the absorption spectrums for the calculated Mg_Al_V_O_ and Mg_Al_ structure. (**b**) Fitting of the absorption spectrums for the calculated MgAl_2_O_4_ and Mg_2_Al_2_O_5_ structures.

To investigate the impact of these structures on the luminescence properties of defect structures, we conducted further research on the optical performance of Mg substitutional doping and the Al-Mg spinel. Given the significant advantages of the GW–BSE (GW approximations plus Bethe–Salpeter equation) method in situations where high-precision optical property predictions are required, we have calculated the absorption spectra of the Mg_Al_ and Mg_Al_V_O_ configurations using the GW–BSE method. The spectra after fitting the peak positions with a Lorentz function are shown in Figure 6a. The absorption spectrum of Mg_Al_ only exhibits characteristic absorption peaks corresponding to the F and F^+^ centers at 191 nm and 253 nm, respectively, which does not align with existing research conclusions that Mg doping affects not only the F and F^+^ centers but also plays a significant role in aggregated defects such as $F_{2}^{+}$ and $F_{2}^{2+}$. In the absorption spectrum of the constructed Mg_Al_V_O_, we can observe not only the F and F^+^ centers but also distinct characteristic peaks associated with aggregated defects. These significant peaks appear at 354 nm ($F_{2}^{+}$) and 434 nm ($F_{2}^{2+}$). This indicates that the Mg_Al_V_O_ configuration more accurately reflects the actual situation of Mg^2+^ doping during crystal growth. The combined effect of Mg^2+^ and oxygen vacancies promotes the generation of aggregated defects. The absorption spectra of the Al-Mg spinel structures obtained using the same computational method are shown in Figure 6b. The MgAl_2_O_4_ structure only shows a characteristic peak of the F center at 192 nm, along with a very small F^+^ absorption peak (as shown in the fitting results of the inset in Figure 6b), while the Mg_2_Al_2_O_5_ structure exhibits absorption peaks similar to those of the Mg_Al_V_O_, also characterizing F and F^+^ centers as well as aggregated defects such as $F_{2}^{+}$ and $F_{2}^{2+}$ centers. Special mention should be made here that, according to the study of the color center of α-Al_2_O_3_, it was shown that the absorption peaks at 228 nm and 243 nm are both characteristic absorption peaks caused by the F^+^ center [5]. This suggests that when considering the doping effect of Mg, the spinel structure influences the property of Mg substitutional doping. The magnitude of this effect is related to the local concentration of Mg in the crystals, and from the present calculations, it appears that the effect is only residual in the case of Mg enrichment.

### 4.2. Experimental Section

**Figure 7:**
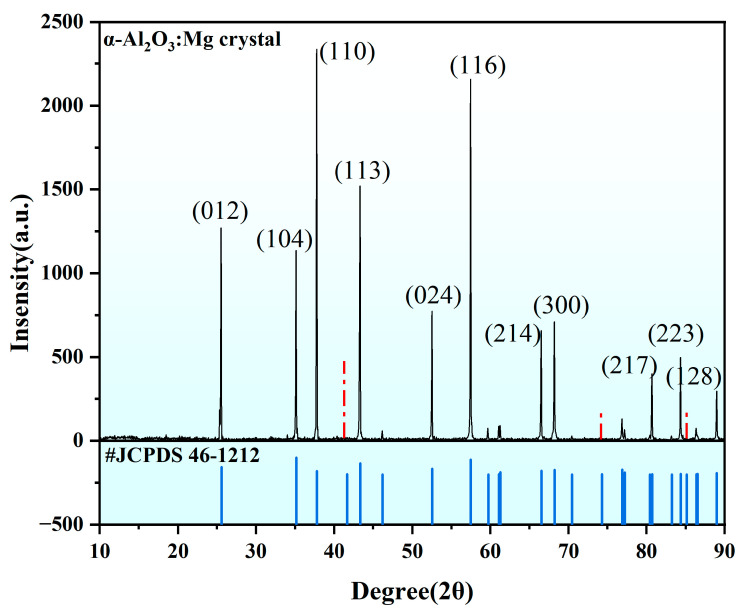
X-ray diffraction of α-A1_2_O_3_:Mg single crystal (measured after grinding single crystals into powder). The difference between the diffraction peaks obtained from the test and the standard card is marked with a red dotted line.

Considering the crystalline systems of our grown Mg-doped Al_2_O_3_. The grown Al_2_O_3_:Mg was ground into powder, and the X-ray diffraction(XRD) pattern obtained from the test is shown in Figure 7. The XRD pattern shows strong diffraction peaks at 25°, 35°, 37°, 46°, and 57°, which are in general agreement with the standard spectrum (JCPDS No. 46-1212 [32]), which suggests that the phase is an α-phase. Since the doping concentration of Mg is 300 ppm, its doping has no obvious effect on the crystal structure, and the absence of diffraction peaks at 41°, 74°, and 85° may be related to the large particle size of the test powder (marked by red dotted lines in the figure).

**Figure 8:**
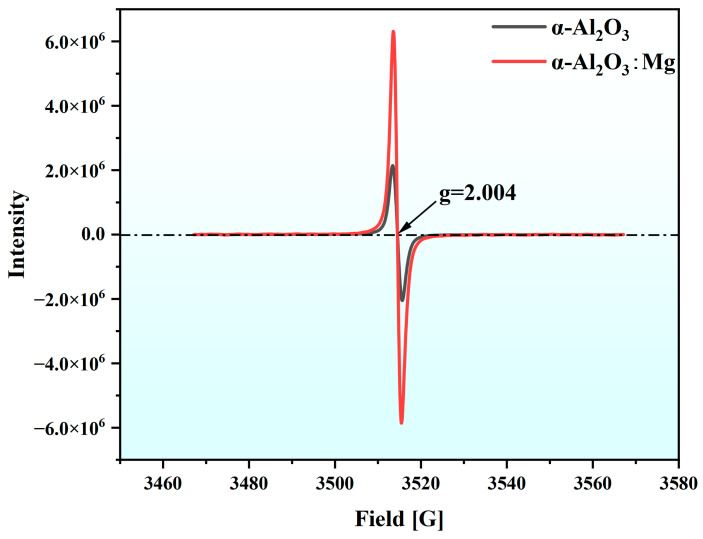
EPR spectrum of α-A1_2_O_3_:Mg single crystal at room temperature.

To confirm the computational results and further explore the impact of local Al-Mg spinel configurations on Mg-doped α-Al_2_O_3_, we conducted a series of tests on the grown α-Al_2_O_3_:Mg crystals and Al-Mg spinel crystals, starting with testing their electron paramagnetic resonance (EPR) spectra, as shown in Figure 8, to justify the existence of metastable charged point structure defects. We deduced that the corresponding g-factor is 2.004. The work of P. A. Kulis et al. [16,17] also obtained consistent g-factors, while their EPR study of the hyperfine structure of Mg-doped α-Al_2_O_3_ crystals after X-ray irradiation (detailed information can be found in the Supplementary Materials of this paper) also showed that the Mg_Al_V_O_ structure we propose is reasonable. At the same time, we compared the EPR spectra of α-Al_2_O_3_:Mg with those of pure Al_2_O_3_ crystals, and it can be clearly seen that the doping of Mg promotes the increase of the defect concentration.

**Figure 9:**
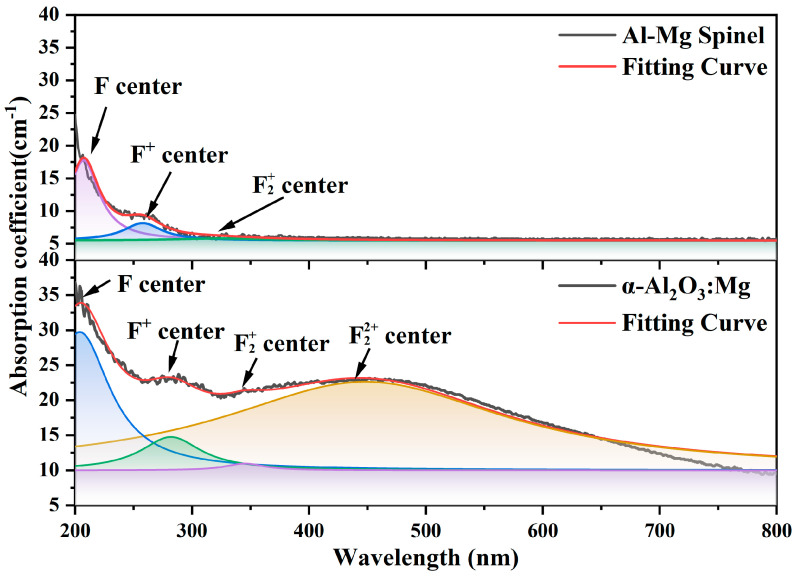
Optical absorption spectra of the as-grown α-Al_2_O_3_:Mg single crystals and Al-Mg spinel.

Absorption spectra and performs Lorentz fitting on their absorption peaks. As shown in Figure 9, the experimentally grown α-Al_2_O_3_:Mg crystals exhibit characteristic peaks corresponding to F, F^+^, $F_{2}^{+}$, and $F_{2}^{2+}$ centers. The fitting curve of the Al-Mg spinel shows characteristic peaks for F and F^+^ centers, as well as a very weak peak for the $F_{2}^{+}$ center, which may be due to the concentration of this Al-Mg spinel single crystal being more in line with the 1:1 ratio of an α-Al_2_O_3_ and MgO configuration. Therefore, it exhibits an absorption curve similar to that of the MgAl_2_O_4_ structure in the calculations. This phenomenon indicates that the spinel structure formed under non-Mg-enriched conditions has a limited impact on the substitutional doping effect of Mg. At the same time, the intensity of the characteristic absorption peaks corresponding to Mg-Al spinel is significantly lower than that of α-Al_2_O_3_:Mg; thus, its influence on the optical performance of α-Al_2_O_3_:Mg crystals can be neglected.

**Figure 10:**
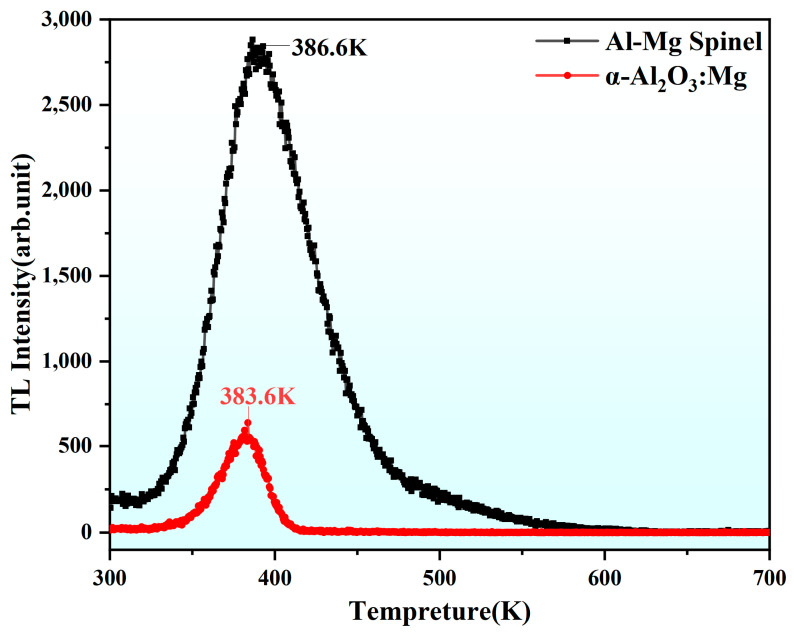
TL curves of the as-grown α-Al_2_O_3_:Mg and Al-Mg spinel samples measured at the heating rate of 5 K/s.

Considering the significant value of the radiation properties of α-Al_2_O_3_:Mg crystals, Figure 10 presents the TL curves for α-Al_2_O_3_:Mg crystals and Al-Mg spinel crystals. Both exhibit a single TL peak, with the peak for α-Al_2_O_3_:Mg crystals at 383.6 K and that for the Al-Mg spinel crystals at 386.6 K. The positions of the TL peaks are very close, and the peak of the Al-Mg spinel is significantly higher than that of α-Al_2_O_3_:Mg. Therefore, for an in-depth study of the role of Mg doping in α-Al_2_O_3_, it is necessary to further consider the impact of its aggregated spinel structure on its TL properties.

## 5. Conclusions

We initially established three models related to Mg^2+^ substituting for Al^3+^ doping: Mg_Al_, V_O_, and Mg_Al_V_O_. The calculated formation energies indicated that the formation of Mg_Al_V_O_ requires less energy than a single Mg substitutional. So, Mg substitutional doping structures incorporating oxygen vacancies are more likely to form in crystals. Comparing the DOS of Mg_Al_ and Mg_Al_V_O_ structures, the single defect Mg_Al_ structure did not generate the gap state in the bandgap, while the Mg_Al_V_O_ structure introduced one occupied energy level and one unoccupied energy level. However, the contribution of Mg to the orbitals is minimal, leading to the analysis that Mg does not directly participate in the formation of gap states; instead, it primarily forms defects by charge compensation to oxygen vacancies. The comparison of DOS between MgAl_2_O_4_ and Mg_2_Al_2_O_5_ structures indicated that when Mg aggregates to form an Al-Mg spinel structure, the resulting Mg enrichment is conducive to the creation of defects within the crystal, promoting the formation of color centers within the structure. The absorption spectrum calculations for Mg_Al_ and Mg_Al_V_O_ structures reflected the rationality of the Mg_Al_V_O_ structure and characterized the absorption peaks corresponding to various color centers induced by Mg doping, including F and F^+^ centers as well as $F_{2}^{+}$ and $F_{2}^{2+}$. The absorption spectra of MgAl_2_O_4_ and Mg_2_Al_2_O_5_ structures verified that the locally Mg-enriched spinel structure (Mg_2_Al_2_O_5_) affects the optical property of α-Al_2_O_3_:Mg crystals.

To validate the computational predictions, we tested the absorption spectra of α-Al_2_O_3_:Mg and Al-Mg spinel crystals and fitted the characteristic peaks of the absorption spectra. The results showed that the absorption peaks of α-Al_2_O_3_:Mg crystals were consistent with the computational results, with distinct characteristic absorption peaks corresponding to F and F^+^ centers as well as $F_{2}^{+}$ and $F_{2}^{2+}$ obtained in the tests. Meanwhile, the fitting curve of the Al-Mg spinel exhibited characteristic peaks for F and F^+^, along with a very weak peak for $F_{2}^{+}$. Additionally, the intensity of the characteristic absorption peaks corresponding to the Al-Mg spinel was significantly lower than that of α-Al_2_O_3_:Mg, thus having a minimal impact on the optical performance of α-Al_2_O_3_:Mg crystals. Finally, both TL properties of α-Al_2_O_3_:Mg and Al-Mg spinel crystals exhibited a single TL peak, with the peak positions being very close. The TL peak of the Al-Mg spinel was significantly stronger than that of α-Al_2_O_3_:Mg. In evaluating the role of Mg doping in α-Al_2_O_3_, it is essential to consider the impact of its aggregated spinel structure on its radiation performance.

## Figures and Tables

**Table 1:** The formation energies of different structures under O-rich conditions.

Structure	Formation Energy (eV)
α-Al_2_O_3_:Mg_Al_	3.368
α-Al_2_O_3_:Mg_Al_V_O_	2.189
α-Al_2_O_3_:V_O_	1.952

**Table 2:** Average mechanical properties of the Mg_Al_V_O_ structure.

Mechanical Properties	Hill’s Algorithm
Bulk Modulus B (GPa)	230.321
Young’s Modulus E (GPa)	346.362
Shear Modulus G (GPa)	138.615
Vickers Hardness (GPa)	16.614

## Data Availability

The original contributions presented in the study are included in the article/Supplementary Materials, further inquiries can be directed to the corresponding author.

## Supplementary Materials

The following supporting information can be downloaded at: https://www.mdpi.com/article/10.3390/ma18020407/s1, Figure S1: Calculated MgO-Al_2_O_3_ phase diagram. The dashed curve is a metastable miscibility gap in MgO; Figure S2: Calculated MgO-Al_2_O_3_ phase diagram. The dashed curve is a metastable miscibility gap in MgO. Reference [33] is cited in the Supplementary Materials.
