# Supplementary material for: The Influence of Mg Doping in α-Al2O3 Crystals Investigated with First-Principles Calculations and Experiment
Source: Materials (Basel). 2025 Jan 16;18(2):407. doi: 10.3390/ma18020407 (PMC11767226; doi:10.3390/ma18020407)
Supplement: Supplementary file 1 [file materials-18-00407-s001.zip › materials-3378818-supplementary.pdf]

Supplementary Materials

The phase diagram of the $MgO-Al_2O_3$ system

We have reviewed the literature on the phase diagram of the $MgO-Al_2O_3$ system as shown in Figure. S1. the alumina crystals grew at a temperature of 2150°C during our experiments, which satisfies the temperature required for the formation of spinel. However, the division of the different components of spinel is not directly written in the phase diagram of the MgO-Al_2O_3 system. Since $Mg_2Al_2O_5$ is formed in the raw material due to localized magnesium-enriched environments so that $Mg_2Al_2O_5$ can appear during the growth process.

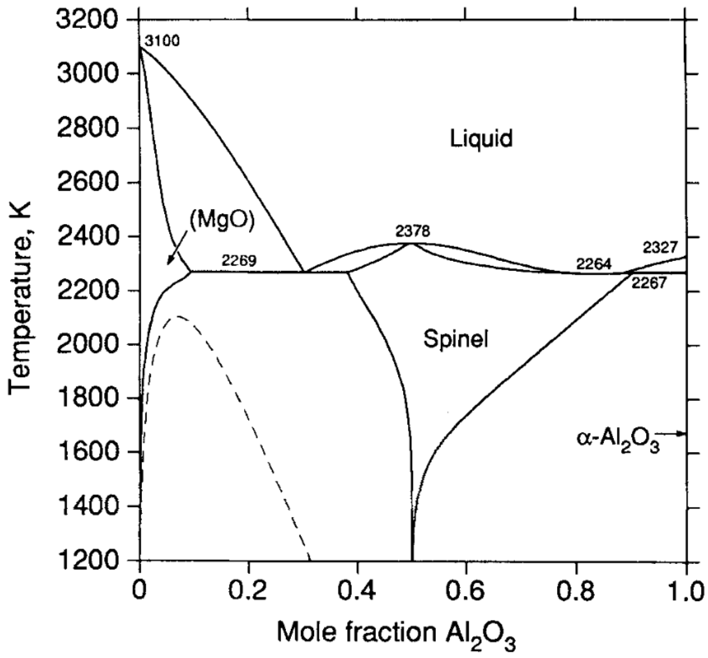

Figure S1. Calculated MgO-Al2O3 phase diagram. The dashed curve is a metastable miscibility gap in MgO. [1]

[1] B. Hallstedt, Thermodynamic Assessment of the System MgO–Al2O3, Journal of the American Ceramic Society **75**, 1497 (1992)

The EPR study of Mg-doped α-Al2O3 crystals after X-ray irradiation

The hyperfine structure of X-ray irradiated Al_2O_3:Mg crystals was studied in more detail in the literature [1]. According to its test results as shown in Figure S2, the X-ray irradiated Mg^{2+} doped crystals exhibit EPR spectra with two centers, and the g-factors of the two color centers were deduced as follows: Centre1: $g_z = 2.005$; $g_x = 2.029$; $g_y = 2.016$; Centre2: $g_z = 2.003$; $g_x = 2.022$; $g_y = 2.019$. The parameters of the first center agree with those of the $[Mg]^0$ center (i.e., $Mg_{Al}V_O$ in our manuscript) obtained by Cox [2] in Mg^{2+}-doped Al_2O_3 crystals. The other center exhibits the hyperfine structure described above due to the fact that Mg^{2+} reduces the symmetry of the crystal when it replaces one Al^{3+} around the vacancy to form the $Mg_{Al}V_O$ structure. The

interaction of electrons with Mg^{2+} around the $Mg_{Al}V_O$ center is no longer equivalent. The electrons mainly interact with the two Al^{3+} away from the Mg ions, resulting in the hyperfine structure observed in the EPR spectra. Thus the test results of EPR in this literature justify the substitution of Mg^{2+} for one Al^{3+} around the vacancy to form the $Mg_{Al}V_O$ structure. The defect aggregates to form an F_2 -type aggregation defect, which shows different color-centered characteristic absorption peaks in the absorption spectra.

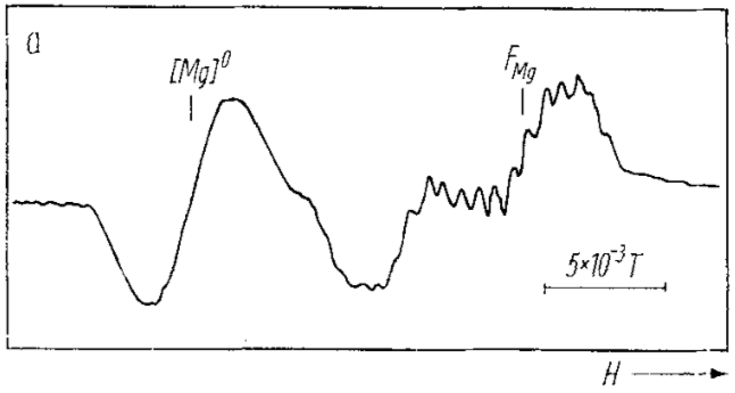

Figure S2. EPR spectrum of Mg-doped α-Al_2O_3 crystal at 80 K after irradiation with X-rays. H in the (1120) plane, the angle between the C_3 axis and H is 60°.

[1] P. A. Kulis, M. J. Springis, I. A. Tale, V. S. Vainer, and J. A. Valbis, Impurity-Associated Colour Centres in Mg- and Ca-Doped Al_2O_3 Single Crystals, Physica Status Solidi (b) **104**, 719 (1981).

[2] R. T. Cox, Electron spin resonance studies of holes trapped at Mg^{2+}, Li^+ and cation vacancies in Al_2O_3., Solid State Communications **9**, 1989 (1971).
